# Neurobiological and therapeutic landmarks of depression associated with Alzheimer’s disease dementia

**DOI:** 10.3389/fnagi.2025.1584607

**Published:** 2025-06-02

**Authors:** Ilinca Untu, Michael Davidson, Gabriela-Dumitrita Stanciu, Jonathan Rabinowitz, Romeo-Petru Dobrin, Diana-Sabina Vieru, Bogdan-Ionel Tamba

**Affiliations:** ^1^Department of Medicine III, Faculty of Medicine, “Grigore T. Popa” University of Medicine and Pharmacy of Iasi, Iași, Romania; ^2^Advanced Research and Development Center for Experimental Medicine “Prof. Ostin C. Mungiu” CEMEX, “Grigore T. Popa” University of Medicine and Pharmacy of Iasi, Iași, Romania; ^3^Bar Ilan University, Ramat Gan, Israel; ^4^Department of Pharmacology, Clinical Pharmacology and Algesiology, “Grigore T. Popa” University of Medicine and Pharmacy of Iasi, Iași, Romania

**Keywords:** Alzheimer’s disease, depression, shared neurobiological mechanisms, depression–Alzheimer’s disease comorbidity, bidirectional relationship

## Abstract

Depression in Alzheimer’s disease (AD) dementia has become an increasingly recognized public health concern due to its high prevalence and substantial impact on patient outcomes. Despite extensive research having been conducted over the past decades, the precise causal mechanisms and the nature of the relationship between depression and AD dementia remain incompletely understood. This narrative review examines the bidirectional interaction between depression and Alzheimer’s disease, emphasizing shared neurobiological pathways, including neurotransmitter dysregulation, neuroinflammation, abnormalities in the hypothalamic–pituitary–adrenal (HPA) axis, and deficits in neuroplasticity. These mechanisms likely contribute to the acceleration of neurodegeneration in AD and the onset or worsening of depressive symptoms. Current therapeutic approaches remain largely nonspecific, with a lack of targeted therapies that address the unique pathophysiological context of depression in AD. While progress has been made, key research gaps remain, particularly in understanding the complex biological interactions between these two conditions. Future research should focus on identifying specific biomarkers and developing personalized treatment strategies tailored to the neurobiological features of both depression and AD. By addressing these neurobiological mechanisms, we can develop more effective and targeted interventions, ultimately improving patient outcomes and advancing clinical care for this dual pathology.

## 1 Introduction: depression in Alzheimer’s: a complex and unseen relationship

A growing body of epidemiological and longitudinal research has revealed a complex interplay between Alzheimer’s disease (AD) and depression. However, whether depression arises as a symptom of neurodegeneration or as a psychological reaction to cognitive impairment remains a subject of debate. Some researchers suggest that depression precedes AD, potentially serving as a risk factor, while others argue that it co-occurs with the disease, acting as an integral component of its progression (Galts et al., 2019; Diniz et al., 2013).

As global populations age, the prevalence of Alzheimer’s disease is projected to rise significantly. Aging remains the most well-established risk factor for AD, and demographic trends indicate a steady increase in the number of older adults worldwide (Hou et al., 2019; Liu et al., 2024). Currently, approximately 5% of individuals over the age of 65 are diagnosed with dementia, with prevalence doubling roughly every 5 years. Estimates suggest that over 55 million people globally are living with some form of dementia, with numbers expected to surge to 78 million by 2030 and 139 million by 2050. The sharpest rise is anticipated in developing nations, where the proportion of dementia cases is projected to grow from the current 60 to 71% by mid-century (Alzheimer’s Association, 2024; Liu et al., 2024).

Alzheimer’s disease is the leading cause of dementia, primarily affecting neurons in brain regions associated with memory, language, executive functioning, and other higher-order cognitive abilities. As a result, early symptoms typically manifest as episodic memory deficits, particularly difficulties in retaining newly acquired information. While these deficits may appear suddenly, the underlying neurodegenerative changes—such as *β*-amyloid deposition and tau pathology—begin nearly two decades before clinical symptoms become evident (Li et al., 2025). As the disease progresses, other cognitive domains become increasingly impaired, including attention, executive function (e.g., planning, decision-making), language (e.g., word-finding difficulties), and visuospatial abilities (e.g., disorientation in familiar places). This gradual deterioration transitions from mild cognitive impairment (MCI) to overt dementia, where patients often retain partial insight into their cognitive decline. This awareness can lead to frustration, anxiety or depression, further complicating the clinical presentation (Coughlan et al., 2018; Stanciu et al., 2020). Ultimately, these cognitive impairments result in a progressive loss of independence, severely affecting daily living and increasing reliance on caregivers.

The diagnosis of Alzheimer’s dementia is typically confirmed when symptoms progress to the point of significantly impairing an individual’s ability to function independently. As a progressive neurodegenerative disorder, AD worsens over time, with the rate of cognitive decline and the specific domains affected varying between individuals. As the disease advances, neuronal damage spreads across multiple brain regions, leading to severe cognitive and functional deterioration. In its later stages, AD leads to a complete loss of independence, requiring full-time care and support from family or caregivers (Langa and Levine, 2014; Coughlan et al., 2018).

Depression, a prevalent affective disorder, disproportionately affects older adults, particularly those receiving medical care, living in institutional settings, or requiring long-term assistance (Alzheimer’s Association, 2024). In late life, it exhibits higher recurrence rates than in middle-aged populations and represents an independent predictor of increased mortality (Mitchell and Subramaniam, 2005). Alarmingly, the risk of suicide is significantly elevated among older adults with depression, with reports indicating that up to 45% of individuals affected by both depression and AD experience suicidal ideation (Olin et al., 2002).

In the context of AD, depression often presents diagnostic challenges. Depression is regarded as a “syndrome” rather than a “disease” due to the absence of a definitive biomarker, making its diagnosis reliant on subjective questionnaire-based assessments. Many neuropsychiatric symptoms associated with depression—such as anxiety, irritability, aggression, and sleep disturbances—are also frequently observed in AD patients (Lyketsos, 2007), complicating differential diagnosis. Although it may share some features with major depressive disorder, depression in AD frequently presents atypically, manifesting as apathy, social withdrawal, or increased irritability (Table 1), rather than pervasive sadness or guilt (Alzheimer’s Association, 2024; Galts et al., 2019). These overlapping and atypical symptoms are often misattributed to the neurodegenerative process itself, which contributes to under recognition and undertreatment. Clinical data suggest that over half of individuals with AD experience comorbid depression, which not only diminishes quality of life but also exacerbates cognitive and functional decline. Depression has been particularly associated with impairments in instrumental activities of daily living (IADLs)—such as managing finances, maintaining social relationships, and performing complex self-care tasks (Giannouli and Tsolaki, 2021). Notably, emerging evidence suggests that depressive symptoms significantly compromise financial decision-making abilities even in patients with mild AD, underscoring the early impact of mood disturbances on executive functioning and autonomy. This cascade of cognitive and functional deterioration contributes to accelerated dependency and heightens caregiver burden (Giannouli and Tsolaki, 2022).

**Table 1:** Comparison of key neuropsychological aspects between depression and Alzheimer’s disease.

Symptom/ Sign	Manifestation in depression	Manifestation in Alzheimer’s disease	Key differences
Cognitive impairment	Difficulty concentrating	Memory loss, particularly short-term memory problems	Memory loss is more prominent in Alzheimer’s
Memory problems	Trouble recalling details or tasks	Short-term memory loss, especially difficulty recalling recent events	Alzheimer’s shows progressive memory loss over time
Affective symptoms	Persistent low mood, feelings of sadness or hopelessness	Emotional blunting, irritability, mood swings	Mood disturbances in depression are more persistent
Fatigue/ low energy	Chronic fatigue, low energy levels	Apathy and reduced motivation, often accompanied by physical fatigue	Apathy in AD is typically linked to cognitive decline
Sleep disturbances	Insomnia or excessive sleepiness, poor sleep quality	Disrupted sleep, especially difficulty staying asleep at night	Sleep issues are more linked to neurodegeneration in AD
Psychomotor changes	Slowed movements, restlessness, agitation	Reduced physical activity, difficulty with coordination	Psychomotor slowing in AD is linked with brain atrophy
Anxiety	Worry, nervousness, restlessness	Anxiety can occur but is often less prominent compared to depression	Anxiety in AD is often secondary to cognitive decline
Social withdrawal	Isolation, avoiding social interactions due to low energy or interest	Social withdrawal, reduced engagement due to cognitive difficulties	Withdrawal in AD often stems from confusion and memory loss
Decision-making problems	Difficulty making decisions, feeling overwhelmed by choices	Impaired decision-making due to cognitive dysfunction	Cognitive decline is a more significant factor in AD
Self-esteem/ self-worth	Feelings of worthlessness, guilt	May express concern or frustration about cognitive decline but with less guilt	AD patients may lack awareness of their cognitive deficits

At the neurobiological level, converging evidence points to shared mechanisms underlying both conditions. Disruptions in neurotransmitter systems—including serotonin, norepinephrine, and dopamine pathways—have been implicated in the pathophysiology of both depression and AD. Neuroinflammation, characterized by elevated pro-inflammatory cytokines, is another key contributor to neuronal damage and mood dysregulation. Additionally, dysregulation of the hypothalamic–pituitary–adrenal (HPA) axis has been observed in both disorders, potentially intensifying cognitive impairment and emotional instability (Zhan et al., 2024; Bajaj and Mahesh, 2024). Deficits in neuroplasticity, particularly within the hippocampus, further reinforce the pathological feedback loop between neurodegeneration and depressive symptoms (Stanciu et al., 2020).

Despite substantial clinical findings, depression associated with Alzheimer’s dementia remains frequently underdiagnosed and undertreated, mostly due to the absence of diagnostic criteria specifically tailored to this complex clinical context. The distinction between early affective disturbances often related to the patient’s awareness of emerging cognitive deficits and clinically significant depressive episodes is often ambiguous. This diagnostic ambiguity can delay or preclude appropriate therapeutic intervention. A key challenge lies in the considerable symptom overlap between depression and the cognitive and functional deterioration characteristic of dementia, compounded by the lack of standardized assessment tools validated for this population (Yun and Kim, 2021; Huang et al., 2024). Beyond neurobiology, increasing evidence points to the critical role of psychosocial and environmental determinants. Stressful life events, such as bereavement, transitions to institutional care, and perceived loss of autonomy, have been implicated as advancing or exacerbating factors in late-life depression, particularly in cognitively vulnerable populations (Giannouli, 2024). Sleep disturbances in particular—such as insomnia, fragmented sleep, or reduced slow-wave sleep—have been linked to cognitive decline not only in individuals with mild cognitive impairment (MCI), but also in those with established Alzheimer’s disease. Emerging evidence implicates altered sleep architecture in impaired memory consolidation, increased *β*-amyloid deposition and decreased lymphatic clearance, all of which may contribute to both neurodegeneration and affective dysregulation (Giannouli and Tsolaki, 2023). Within the broader category of lifestyle interventions, adherence to dietary models rich in neuroprotective and anti-inflammatory components has been consistently associated with a lower risk of both depressive disorders and neurodegenerative decline (Al Shamsi et al., 2024). Specifically, the Mediterranean (MeDi), Dietary Approaches to Stop Hypertension (DASH), and Mediterranean-DASH Intervention for Neurodegenerative Delay (MIND) diets have demonstrated favorable associations with cognitive preservation and emotional well-being. The MeDi and DASH diets emphasize high consumption of vegetables, fruits, whole grains, legumes, nuts, fish, and unsaturated fats (particularly from olive oil), while limiting intake of red meat, saturated fats, refined sugars, salt and alcohol (Tangney et al., 2014; Wengreen et al., 2013). The MIND diet, a hybrid of the MeDi and DASH frameworks, uniquely specifies olive oil as the principal source of dietary fat and prioritizes berry consumption as the sole fruit category, based on their high polyphenolic content. Nutritional components integral to these diets—such as omega-3 polyunsaturated fatty acids (n-3 PUFAs), flavonoids and other polyphenols—have been implicated in the maintenance of synaptic plasticity, reduction of oxidative stress, and modulation of neuroinflammation, all of which are processes relevant to both mood regulation and neurodegeneration in aging populations (Al Shamsi et al., 2024).

Collectively, these multifactorial influences, ranging from molecular alterations to psychosocial and behavioral factors, call for a comprehensive integrative framework. This narrative review aims to explore and present the current evidence on the complex bidirectional relationship between depression and Alzheimer’s disease. A more nuanced understanding of these overlapping and converging pathways may inform more precise diagnostic strategies and the development of targeted therapeutic strategies for patients suffering from both AD and comorbid depression.

## 2 Neurobiological mechanisms shared between depression and Alzheimer’s disease

**Figure 1:**
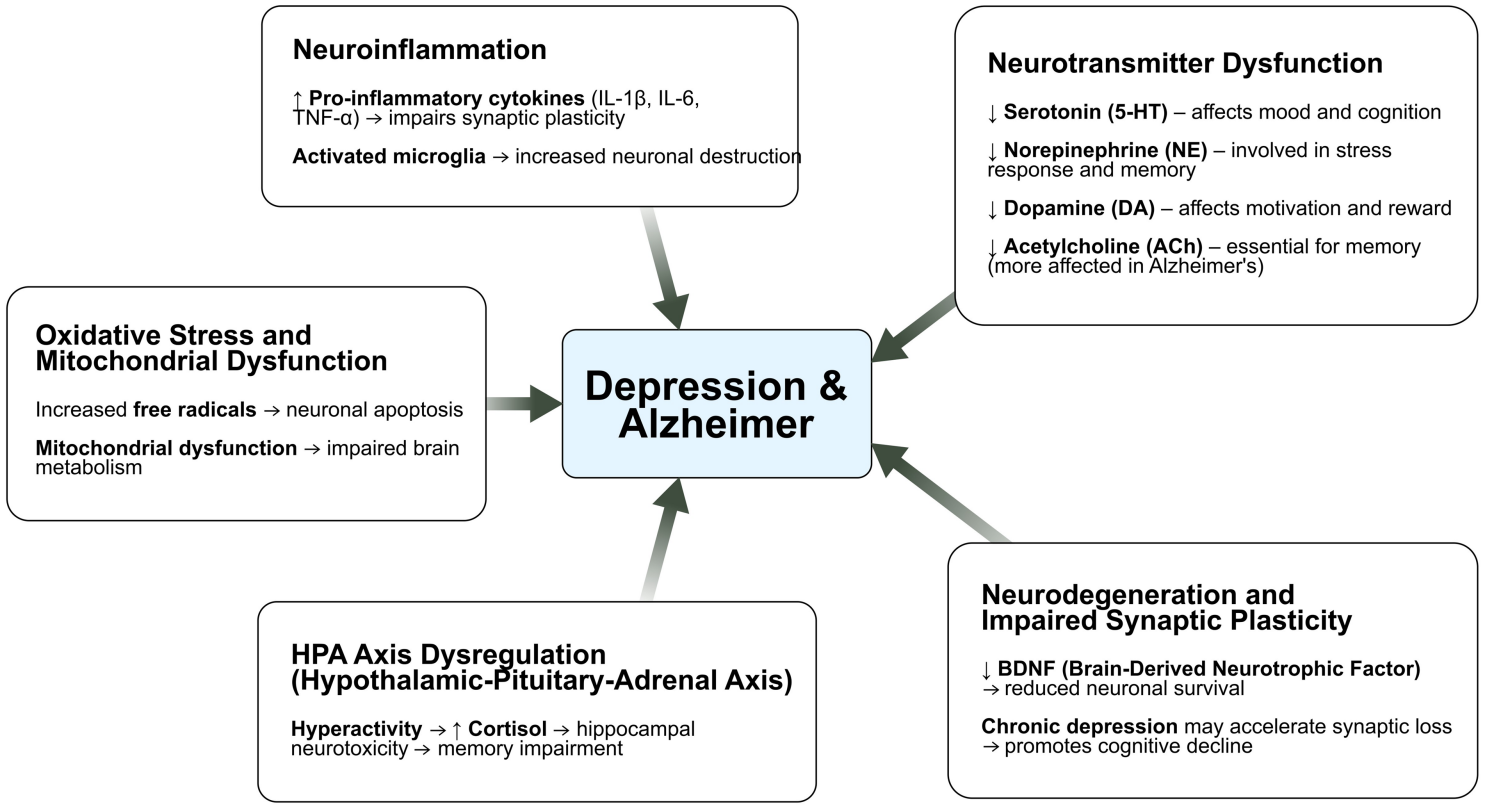
Shared mechanisms across depression and Alzheimer’s disease - key risk factors underlying their association. These include genetic predisposition, neurovascular dysfunction, impaired hippocampal neurogenesis, structural brain changes, and dysregulation of the cortisol-hippocampal pathway. Genetic factors may increase susceptibility to both disorders by influencing neuroinflammation, synaptic plasticity, and neuronal resilience. Neurovascular changes, such as reduced cerebral blood flow and blood–brain barrier disruption, may exacerbate neurodegeneration and mood disturbances. Impaired hippocampal neurogenesis and structural alterations, particularly in regions linked to memory and emotion regulation, further strengthen the connection between the two conditions. Finally, chronic activation of the hypothalamic–pituitary–adrenal (HPA) axis and elevated cortisol levels can accelerate hippocampal atrophy, a shared pathological feature of depression and Alzheimer’s disease. These interconnected mechanisms highlight potential targets for early detection and therapeutic interventions.

Depression and Alzheimer’s disease share a complex, bidirectional relationship, underpinned by overlapping neurobiological mechanisms. Shared risk factors— including genetic, environmental, structural and neurochemical—may contribute to their frequent co-occurrence (Figure 1). The heterogeneity of depression characterized by diverse symptomatology and subtypes, may influence the variability in disease progression and association with Alzheimer’s pathology. These overlapping pathophysiological substrates not only help explain the frequent comorbidity of depression and Alzheimer’s disease but also point to potential shared biomarkers that could support earlier identification and differential diagnosis.

Clinical and neuropathological studies have demonstrated that Alzheimer’s patients with coexisting depression score lower on cognitive assessments, such as the CAMCOG test, suggesting that depressive symptoms may accelerate neurodegeneration (Milwain and Nagy, 2005; Huppert et al., 1995). Post-mortem studies further support this, revealing nearly double the number of amyloid plaques and neurofibrillary tangles in the hippocampus AD patients with comorbid depression compared to those without (Rapp et al., 2006). Longitudinal studies have also shown that depression may precede and even predict the onset of dementia, underscoring its potential role in accelerating neurodegeneration (Brunnström et al., 2013).

In addition to its association with cognitive decline, depression is a well-established risk factor for Alzheimer’s, even when the two conditions are separated by decades, a finding supported by a meta-analysis conducted by Ownby et al. (2006). Moreover, depression is highly prevalent among Alzheimer’s patients—affecting up to 75%, and up to 90% of female patients (Usman et al., 2010). This is particularly important, as depression not only worsens amyloid pathology but also accelerates the clinical progression of Alzheimer’s disease, contributing to faster cognitive decline and greater functional impairment (Zahodne et al., 2015).

### 2.1 Shared genetic mechanisms

Recent genetic research highlights significant commonalities between depression and AD, supporting the hypothesis of a shared genetic basis for both disorders. A recent genome-wide association study (GWAS) leveraging data from the UK Biobank identified 98 shared genetic variants between the two conditions, with TMEM106B emerging as a significant candidate. This gene has been linked to both depression and AD, as well as structural brain differences, including alterations in the corpus callosum and temporal cortex, suggesting a genetic bridge between affective symptoms and neurodegenerative changes (Monereo-Sánchez et al., 2021).

Among neurotrophic factors, brain-derived neurotrophic factor (BDNF) has received considerable attention due to its role in synaptic plasticity, neuronal survival, and mood regulation. Polymorphisms such as G196A (Val66Met) are more prevalent in AD patients with comorbid depression and have been associated with both the severity of depressive symptoms and response to antidepressant treatment (Zhang et al., 2011; Borroni et al., 2009; Arlt et al., 2013). In contrast, another SNP within the BDNF gene, C270T, does not appear to contribute significantly to depression in AD (Borroni et al., 2009; Grunblatt et al., 2009). Although BDNF is known to play a central role in depression, relatively few studies have explored its expression in AD patients with comorbid depressive symptoms. One such study found no significant differences in cerebrospinal fluid (CSF) BDNF levels between depressed and non-depressed AD patients (Pláteník et al., 2014). Beyond genetic polymorphisms, disruptions in BDNF signaling itself may contribute to Alzheimer’s pathology in a bidirectional manner. Impaired BDNF activity has been shown to facilitate the upregulation of amyloidogenic proteins such as amyloid precursor protein (APP) and presenilin-1 (PS1), leading to increased amyloid-*β* (Aβ) production in transgenic mice (Matrone et al., 2008). Conversely, Aβ has been demonstrated to inhibit neuroprotective signaling cascades—including the Raf-MAPK/ERK and PI3K-Akt pathways—by interfering with the interaction between BDNF and its receptor TrkB, resulting in synaptic dysfunction and neuronal stress (Tong et al., 2004). Notably, recent research using the 5xFAD mouse model of AD has shown that microglial repopulation restores BDNF expression and reactivates TrkB-mediated pathways, significantly improving synaptic plasticity and cognitive outcomes (Wang S. M. et al., 2023; Wang W. et al., 2023). To complement the role of BDNF, other neurotrophic factors have also been implicated in AD-related depression. For example, the CC genotype of transforming growth factor-beta 1 (TGF-β1) has been associated with an increased risk of depressive symptoms in AD patients (Caraci et al., 2012).

Other genetic contributors have also been implicated. The choline O-acetyltransferase (CHAT) gene, essential for cholinergic transmission, has been linked to depressive symptoms in AD, particularly in individuals carrying the ApoE ε4 allele—suggesting an interaction between cholinergic vulnerability and amyloid burden (Grunblatt et al., 2009). In contrast, genes related to dopamine transport, such as DAT1, have not shown consistent associations with depression in AD (Proitsi et al., 2012).

Emerging research has also highlighted the role of Sirtuin 2 (SIRT2) in neurodegeneration and affective disorders. The rs10410544 TT genotype appears to exert a protective effect against depression in AD (Porcelli et al., 2013), and experimental studies in aging mouse models have shown increased hippocampal SIRT2 protein expression with age, independent of mRNA levels—suggesting post-translational accumulation. Pharmacological inhibition of SIRT2 has led to cognitive improvement and reduced neuroinflammation in younger mice, although its effectiveness diminishes in more advanced aging stages (Diaz-Perdigon et al., 2020). Functional assays in SH-SY5Y cells further demonstrated that SIRT1 exerts a neuroprotective effect, counteracting Aβ42-induced toxicity, while SIRT2 exacerbates neuronal vulnerability, reducing cell survival (Li N. et al., 2023; Li Z. et al., 2023).

Recent findings indicates that late-life depression, particularly when it precedes the onset of AD by several years, may act as an independent risk factor for dementia. This association is especially strong in non-carriers of the ApoE ε4 allele, where depression may represent an independent contributor to neurodegeneration rather than a prodromal symptom (Sun et al., 2009). Animal models carrying mutations in the APP, in combination with human ApoE isoforms, confirm that the presence of the ApoE ε4 allele significantly enhances Aβ accumulation, largely through impaired clearance mechanisms, further substantiating the genetic interplay between mood disorders and AD pathophysiology (Bales et al., 2009; Castellano et al., 2011). Consistently, both neuropathological studies and neuroimaging in humans have revealed that ApoE ε4 carriers develop more extensive and earlier Aβ pathology than non-carriers, reinforcing the notion of genotype-specific vulnerability (Morris et al., 2010).

### 2.2 Neurotransmitter dysregulation

The serotonergic system itself bridges depression and AD due to serotonin’s critical role in mood regulation and cognitive function. Serotonin has seven families of receptors (5-HT1–7), which are all G-protein-coupled except for the ionotropic 5-HT3 receptor (Grunblatt et al., 2009; Micheli et al., 2006; Pritchard et al., 2007; Proitsi et al., 2012; Liu et al., 2001; Mohammad-Zadeh et al., 2008). The most prescribed antidepressants, selective serotonin reuptake inhibitors (SSRIs), target serotonin restoration in the central nervous system. SSRIs are foundational in the serotonin hypothesis of depression, which attributes depression and its complications to reduced serotonergic neurotransmission (Fakhoury, 2016). Besides the serotonin transporter (SERT), three serotonin receptor subtypes (5-HT1A, 5-HT1B, and 5-HT2A), predominantly localized in the limbic system, are particularly important in depression. In AD, serotonin and its metabolites are found in reduced quantities in frontal and temporal cortices, highlighting a potential link to the disease’s pathology. Amyloidogenic activity also increases in the post-menopausal period, possibly due to decreased serotonergic signaling stemming from lower estrogen levels, which is consistent with the higher prevalence of AD in women (Bethea et al., 2017). The expression of 5-HT1A receptors, particularly in the hippocampus, is reduced in Alzheimer’s patients, correlating with worsened clinical symptoms (Kepe et al., 2006; Lanctôt et al., 2007). Similarly, 5-HT2 receptors decrease by up to 69%, documented by reduced binding of 5-HT2 ligands, such as setoperone and altanserin, and corresponding with cognitive deficits (Blin et al., 1993). Moreover, recently discovered serotonin receptors, 5-HT4 and 5-HT6, play critical roles in depression and AD pathophysiology. 5-HT4 receptor activation has been shown to have an antidepressant-like effect and restore cognitive abilities in depression (Mendez-David et al., 2014; Darcet et al., 2016). Conversely, 5-HT6 receptor inhibition has been associated with antidepressant-like effects and could help improve memory and learning deficits in Alzheimer’s patients (Geldenhuys and Van der Schyf, 2011). Further supporting this, experimental studies have demonstrated that pharmacological targeting of 5-HT6 receptors can attenuate AD-related pathology. In an AD mouse model, the 5-HT6 antagonist SB271036 reversed memory impairments, likely through *γ*-secretase inhibition—leading to reduced amyloid-beta accumulation—and through the suppression of glial cell activation (Yun et al., 2015). The compound also restored serotonin levels, potentially via indirect GABAergic modulation of serotonergic neurons. Moreover, SSRIs such as fluoxetine have shown neuroprotective and cognition-enhancing effects in both *in vitro* and *in vivo* models of AD. In a familial AD model, cells expressing the Swedish APP mutation demonstrated altered responsiveness of the serotonergic system, particularly involving the 5-HT1B receptor (Tajeddinn et al., 2016). In double transgenic AD mouse models, fluoxetine prevented neuronal loss, improved spatial memory, and delayed synaptic deterioration (Ma et al., 2017; Zhou et al., 2018).

Other neurotransmitter systems, including the dopaminergic and cholinergic pathways, have also been explored in the context of AD-related depression. While one study found no significant association between the dopamine D4 receptor (D4DR) polymorphism and depressive symptoms in AD patients (Grunblatt et al., 2009), a larger cohort study involving over 1,000 individuals with AD identified a significant correlation between the dopamine receptor D3BalI polymorphism and depression. These findings underscore a potential role for the dopaminergic system in the neuropsychiatric manifestations of Alzheimer’s disease. Expanding on these findings, preclinical research using the TgF344-AD rat model has revealed early alterations in serotonergic–dopaminergic connectivity. At 6 months of age, prior to amyloid plaque accumulation, these animals exhibited reduced striatal dopamine release following 5-HT2A receptor antagonism, despite unaltered receptor binding, indicating functional disconnection rather than receptor loss. Enhanced sensitivity of both postsynaptic D2 receptors and presynaptic D2 autoreceptors was also observed in the absence of changes in receptor density or neuroinflammation markers. Interestingly, administration of the SSRI citalopram modified 5-HT2A–D2 receptor functional dynamics without affecting D2 autoreceptors hypersensitivity. In aged animals, amyloid pathology and increased translocator protein expression were found in dopaminergic regions, coinciding with decreased 5-HT2A receptor density in the nucleus accumbens and substantia nigra, likely mediated by astrocytic receptor expression (Ceyzériat et al., 2021).

Cholinergic dysfunction is a well-established feature of Alzheimer’s disease (AD), where impaired cholinergic signaling is believed to contribute to learning and memory deficits—an effect that can be experimentally reproduced using anticholinergic drugs. The cholinergic hypothesis of AD posits that cognitive impairments largely stem from reduced cholinergic transmission, a theory supported by the partial efficacy of acetylcholinesterase inhibitors in alleviating symptoms in some patients (Craig et al., 2011). However, the limited response observed in a subset of individuals complicates this framework and suggests additional mechanisms may be involved. In depression, the cholinergic system also plays a crucial, albeit more complex, role. Whereas diminished cholinergic activity is linked to cognitive decline in AD, evidence points toward cholinergic hyperactivity in depression (Dilsaver and Coffman, 1989). Over recent decades, research has suggested that excessive cholinergic tone may underlie depressive symptoms (Bassi et al., 2015). For instance, antagonizing nicotinic receptors can produce antidepressant-like effects, while cholinergic overactivation is associated with depressive behaviors. Interestingly, activation of specific nicotinic receptor subtypes—particularly the α7 receptor—has been shown to alleviate depressive symptoms by enhancing hippocampal function (Zhao et al., 2017). These findings suggest that the cholinergic system exerts divergent effects in AD and depression, with the hippocampus playing a central modulatory role. Expanding on this, recent findings indicate that Aβ1–42 forms a high-affinity, stable complex with the α7 nicotinic acetylcholine receptor (α7 nAChR) *in vitro* and co-localizes with neuritic plaques in the AD brain, suggesting a direct modulatory effect on cholinergic neurotransmission (Wang et al., 2000). In the PDAPP transgenic mouse model of AD with microdialysis revealed significant Aβ-dependent disruptions in both basal and evoked acetylcholine (ACh) release compared to wild-type controls. These deficits were associated with Aβ interactions not only with α7 nAChRs but also with the high-affinity choline transporter, potentially impairing both steady-state and activity-dependent ACh release. Notably, administration of the anti-Aβ antibody m266 fully restored hippocampal ACh release and choline uptake, while also improving habituation learning—a behavioral deficit typical of this model. These results suggest that soluble Aβ species can directly induce cholinergic dysfunction and cognitive impairment in early AD, even in the absence of overt neurodegeneration. Anti-Aβ immunotherapy may therefore represent a promising approach for the rapid reversal of early cholinergic deficits (Bales et al., 2006).

### 2.3 Hypothalamic–pituitary–adrenal axis dysregulation and stress response

A pathophysiological link between depression and dementia could be mediated by dysregulation of the hypothalamic–pituitary–adrenal (HPA) axis (Caraci et al., 2010; Krishnan and Nestler, 2008; Crochemore et al., 2005; Galts et al., 2019). Studies comparing cortisol levels in individuals with depression or dementia to a control group show that those with both dementia and comorbid depression exhibit the highest cortisol levels. Additionally, in patients with both conditions, cortisol levels are correlated with cognitive function, suggesting an impact on cognitive decline (Barca et al., 2019). However, HPA dysfunction in AD may develop as the disease progresses, and at present, plasma cortisol concentrations do not serve as a reliable biomarker to distinguish between depression and dementia (Galts et al., 2019; Chi et al., 2014).

The dysfunction of the HPA axis, leading to excessive glucocorticoid release, has been observed in both chronic depression and AD patients. Elevated glucocorticoid levels in plasma and CSF are commonly found in both conditions (Popp et al., 2009). However, the pattern of hypercortisolemia differs between the two: in AD, cortisol elevation is phasic, whereas in depression, it tends to be chronic. Further evidence suggests distinct pathophysiological mechanisms underlying depression in AD compared to idiopathic depression (Notarianni, 2013). Studies examining CSF cortisol levels in depressed and non-depressed AD patients found no significant differences, although cortisol levels in AD patients were notably higher than in controls (Hoogendijk et al., 2006). Similarly, plasma cortisol levels in AD patients, with or without depressive symptoms, showed no significant variation, reinforcing the idea of a different underlying mechanism of depression in AD compared to primary depression (Zverova et al., 2013).

In young 3xTg-AD mice, an activated HPA axis is observed during the early stages of neuropathology, despite normal glucocorticoid levels. This activation is detectable even when the mice exhibit only mild behavioral alterations, indicating that neuroendocrine regulation occurs prior to the onset of severe AD-like pathology and more pronounced behavioral deficits. Furthermore, when these animals undergo periodic cognitive stimulation, both corticosterone levels and Aβ pathology increase. These findings suggest that dysfunction in the HPA axis may transform typically stimulating environments into stress-inducing ones, thus contributing to a feedback loop that promotes Aβ release and plaque formation (Sharan and Vellapandian, 2024).

### 2.4 Neuroinflammation and vascular contributions to neurodegeneration

Vascular risk factors, such as hypertension, diabetes, cardiovascular diseases, and stroke, have been linked to both late-life depression and AD (Regan et al., 2006). Many AD patients exhibit mixed types of vascular dementia, and cerebrovascular disease has been suggested to contribute to a form of depression known “Vascular Depression.” This form of depression is linked to several mechanisms, including brain circuit disconnections, inflammation and hypoperfusion, which are interrelated and complementary (Taylor et al., 2013). Studies indicate that a history of stroke is associated with an increased risk of depression and apathy in AD patients, possibly due to disrupted neural circuitry in brain regions involved in mood regulation (Treiber et al., 2008). Moreover, hypertension and asymptomatic stroke have been found to exacerbate depressive symptoms in AD, suggesting the role of cerebrovascular damage in mood disorders (Moon et al., 2013). Additionally, spontaneous cerebral emboli, which damage the fronto-striatal pathways, have been linked to clinically relevant depressive symptoms in patients with both AD and vascular dementia (Purandare et al., 2006). These findings suggest that ischemia and white matter lesions may disrupt neural connections, contributing to depression in AD patients, thus supporting the vascular depression theory. However, some studies have not found vascular risk factors to modify depression in AD, which raises controversy on the exact nature of their relationship (Steinberg et al., 2014; Luchsinger et al., 2008).

On a different note, neurovascular dysfunction in mood-regulating brain regions, associated with increased blood–brain barrier (BBB) permeability, may serve as a link between chronic stress and depression. This dysfunction may involve the VEGF/VEGFR2 pathway (Matsuno et al., 2022) abnormal blood vessel morphology, and peripheral cytokine infiltration into the brain (Menard et al., 2017). Research has shown that the deletion of Lrp1 in brain capillaries exacerbates AD pathology and cognitive impairment (Storck et al., 2016; Nation et al., 2019). Interestingly, BBB breakdown in the hippocampus does not appear to be directly related to Aβ or tau biomarkers in individuals with early cognitive decline, highlighting the complexity of the relationship between vascular dysfunction and AD pathology (Nation et al., 2019). Further contributing to neurovascular compromise, reduced activity and expression of the glucose transporter GLUT-1 at the BBB suggests impaired glucose uptake and utilization by the brain. This metabolic deficiency may aggravate cerebrovascular degeneration and further promote BBB disruption. In animal models overexpressing APP, GLUT-1 dysfunction has been linked to intensified Aβ deposition, neurodegeneration, and the emergence of cognitive deficits (Winkler et al., 2015). Microglia detect Aβ deposits through surface receptors, including Toll-like receptors (TLRs) and receptors for advanced glycation end-products (RAGE). Activation of these receptors triggers an inflammatory cascade designed to eliminate toxic components. This process underscores the dual nature of microglial activation, which can either mitigate or exacerbate amyloid pathology depending on the stage of the disease (Wu et al., 2021). During the phagocytosis of Aβ, microglia release several pro-inflammatory cytokines, including interleukin-1 beta (IL-1 beta), tumor necrosis factor-alpha (TNF-a), interleukin-6 (IL-6), which promote local inflammation, cause neurodegenerative effects and contribute to synaptic disfunction. This prolonged process leads to production of reactive oxygen species (ROS) and nitric oxide (NO), which increase oxidative stress and neuronal damage, contributing to the indirect pathway of neuronal loss and impaired synaptic function (Wu et al., 2021).

Inflammation is a common feature of multiple psychiatric disorders. In depression, the Maastricht study has found that elevated serum biomarkers of inflammation such as the acute phase reactant C-reactive protein (CRP) and TNF-a were associated in both major and minor depressive episodes (van Dooren et al., 2016). Inflammation in depression may be linked to altered connectivity between brain regions. A resting state MRI study on 48 medication-free patients with major depression revealed serum levels of CRP were associated with reduced connectivity between the ventral striatum and prefrontal cortex (correlating with increased anhedonia), the dorsal striatum and prefrontal cortex (correlating with psychomotor slowing). Inflammatory cytokines like IL-6, IL-1 beta and IL-1 receptor antagonist were also linked to diminished connectivity between the striatum and prefrontal cortex (van Dooren et al., 2016). A longitudinal MRI analysis conducted on transgenic J20 mice, compared to wild-type littermates, identified disruptions in neural networks of J20 mice, particularly between posterior and anterior brain regions, with age-related exacerbation of these changes. Reduced fractional anisotropy in the corpus callosum of these AD mouse models revealed white matter microstructural changes, correlated with axonal degeneration and myelin disruption associated with AD, which may be detectable in the pre-symptomatic stages. The paper highlights key features of the disconnection syndrome hypothesis in AD, in which the progression of symptoms is related to functional and/ or structural disconnection between regions, rather than localized changes in specific isolated brain areas (Magalhães et al., 2024).

Alterations in white matter are frequently observed in patients with depression, potentially underlying the dysfunction of fronto-cortico-limbic neural circuits that are crucial to the pathogenesis of depression, especially in the context of emotion dysregulation symptoms (Price and Drevets, 2010; Buckner et al., 2008). In comparison to healthy controls, individuals with major depression exhibited increased functional connectivity (FC) in the anterior default mode network (DMN) and between the anterior DMN and the salience network (Wang S. M. et al., 2023; Wang W. et al., 2023), while showing decreased FC in the posterior DMN and between the posterior DMN and the central executive network. This pattern of dissociation could be attributed to a reduction in the integrity of white matter within the cingulate bundle, which connects the two regions (Charlton et al., 2014). In patients with late-onset depression, abnormal FC was found to correlate not only with the severity of depression but also with the accumulation of cerebral Aβ (Wang et al., 2021) and generalized cognitive dysfunction. Changes in connectivity within the DMN may offer a potential neural mechanism linking depression, Aβ pathology and the progression of Alzheimer’s disease (Yin et al., 2015; Guan et al., 2022).

Despite the complexity and ongoing controversies, these findings, as summarized in Table 2, emphasize the need for an integrated approach to understanding depression in AD. Future research should focus on elucidating the precise molecular and neurobiological mechanisms that link these conditions, as well as identifying biomarkers that could facilitate early diagnosis and targeted interventions. Clinically, the recognition of depression as an early marker or comorbid condition in AD may offer opportunities for more effective treatment strategies that address both mood symptoms and cognitive decline. By targeting modifiable risk factors, such as vascular health and neurotrophic support, interventions may help slow AD progression and improve the quality of life for affected individuals.

**Table 2:** Summary of shared neurobiological mechanisms between depression and Alzheimer’s disease and their clinical implications.

Shared mechanism	Key components/ pathways	Clinical implications
Neurotrophic dysfunction	↓ BDNF, TrkB signaling; polymorphisms in BDNF (e.g., Val66Met), ↓TGF-β1	Synaptic dysfunction, impaired neuroplasticity, cognitive deficits, treatment resistance
Genetic overlap	shared SNPs (e.g., TMEM106B), GWAS-identified risk loci	Increased vulnerability to both disorders; shared heritable risk factors
Neuroinflammation	microglial activation, ↑pro-inflammatory cytokines (IL-1β, IL-6, TNF-α), ROS, NO production	Synaptic damage, neuronal death, progression of neurodegeneration and mood symptoms
Vascular dysfunction	BBB breakdown, ↓GLUT-1 expression, VEGF/VEGFR2 pathway dysfunction	Impaired cerebral perfusion, increased Aβ deposition, heightened neuroinflammation
Amyloid and tau pathology	Aβ accumulation, interaction with BDNF signaling, tau phosphorylation	Cognitive impairment, mood dysregulation, progression of AD pathology
HPA axis dysregulation	↑cortisol levels, hippocampal atrophy, GR signaling imbalance	Emotional regulation deficits, memory impairment, exacerbation of depressive and AD symptoms
Neurotransmitter dysregulation	↓serotonin, norepinephrine, dopamine; impaired GABA/glutamate balance	Depressive symptoms, altered cognition, impaired reward/motivation processing

↓, decreased; ↑, increased; Aβ, amyloid-beta; APP, amyloid precursor protein; BBB, blood–brain barrier; BDNF, brain-derived neurotrophic factor; GR, glucocorticoid receptor; GWAS, genome-wide association study; HPA axis, hypothalamic–pituitary–adrenal axis; IL-1β, interleukin-1 beta; NO, nitric oxide; ROS, reactive oxygen species; SNP, single nucleotide polymorphism; TGF-β1, transforming growth factor-beta 1; TNF-α, tumor necrosis factor-alpha; TrkB, tropomyosin receptor kinase; VEGF, vascular endothelial growth factor; VEGFR2, vascular endothelial growth factor receptor 2.

## 3 Therapeutic strategies for depression associated with AD

Depression is commonly observed in individuals with AD, and strategies for managing this condition remain a subject of ongoing debate. The challenge of diagnosing depression in the context of dementia, given the overlap between depressive and cognitive symptoms, underscores the need for personalized and effective therapeutic approaches.

Current treatment guidelines recommend antidepressants as first-line therapy for depression associated with AD, prioritizing medications with minimal side effects. Among the most commonly prescribed options are Selective Serotonin Reuptake Inhibitors (SSRIs) and Serotonin-Norepinephrine Reuptake Inhibitors (SNRIs), frequently cited in the literature (Bartels et al., 2018). In recent years, several systematic reviews and meta-analyses have investigated the efficacy of these treatments in AD-related depression. While findings suggest potential benefits, results remain limited and sometimes contradictory (Hsu et al., 2021; Sanchez et al., 2015; Huang et al., 2022; Li N. et al., 2023; Li Z. et al., 2023) (Table 3). One systematic review and meta-analysis focusing on serotonergic antidepressants (Hsu et al., 2021) found that these agents significantly improved neuropsychiatric symptoms (NPS) and agitation (with small effect sizes), alleviated depressive symptoms, reduced care burden, and slightly enhanced cognitive function. Subgroup analyses revealed that both SSRIs (particularly citalopram/ escitalopram) and non-SSRIs effectively reduced agitation and depressive symptoms, though only SSRIs showed broader effects on NPS and care burden. Furthermore, benefits were more pronounced among patients with AD-related depression compared to those with pure AD. These medications were generally well-tolerated, with no significant differences in attrition rates or adverse events compared to placebo (Hsu et al., 2021). In addition to serotonergic agents, vortioxetine, a newer antidepressant with multimodal pharmacological actions, has been evaluated in two recent meta-analyses. Vortioxetine was shown to significantly improve cognitive function and depressive symptoms, with greater effects at higher doses, as measured by the Montgomery-Åsberg Depression Rating Scale (MADRS). These findings suggest that vortioxetine may support cognitive recovery even when depressive symptoms persist. However, its potential role in preventing dementia requires further longitudinal investigation (Huang et al., 2022; Li N. et al., 2023; Li Z. et al., 2023). In clinical practice, vortioxetine appears to be effective, well-tolerated, and safe, contributing to improved cognitive and functional outcomes.

**Table 3:** The interplay between depression treatment and Alzheimer’s disease progression: insights from pharmacological and psychological therapies.

Topic	Pharmacological interventions	Psychological interventions
Antidepressant therapy	*SSRIs (Selective Serotonin Reuptake Inhibitors):* are supported by high-level evidence from meta-analyses and randomized controlled trials (RCTs). - Increase serotonin levels, potentially reducing neuroinflammation and oxidative stress, both of which are associated with the pathogenesis of Alzheimer’s (Bartels et al., 2018; Cirrito et al., 2020; Sheline et al., 2020). - Some studies suggest a potential reduction in the risk of AD onset in patients with depression treated with SSRIs. Long-term use may improve brain plasticity, delaying cognitive decline (Hsu et al., 2021; Sanchez et al., 2015; Huang et al., 2022; Li N. et al., 2023; Li Z. et al., 2023) - Potential for side effects such as sexual dysfunction, nausea, and increased risk of fall (Orgeta et al., 2017; Dudas et al., 2018). *Tricyclic Antidepressants (TCAs):* found to have moderate-level evidence from reviews and meta-analyses - Inhibit serotonin and norepinephrine reuptake, with possible neuroprotective effects. - Long-term use in older adults may increase the risk of cognitive decline due to anticholinergic side effects (Modrego, 2010; Thompson et al., 2007). *SNRIs (Serotonin-Norepinephrine Reuptake Inhibitors):* shown to have moderate-level evidence based on meta-analyses - Elevate both serotonin and norepinephrine, which may help in neuroprotection, preventing the onset of AD. - Some evidence of reduced AD risk when treated for depression with SNRIs (He et al., 2021). *Mirtazapine:* evidence suggests it has moderate-level support based on clinical trials and meta-analyses - Atypical antidepressant with sedative properties, useful in patients with AD and comorbid depression or insomnia. - Improves sleep, appetite, and mood in AD patients. Sedation and weight gain (He et al., 2021).	*Cognitive behavioral therapy (CBT):* Level of evidence: high - Identifies and modifies negative thought patterns and maladaptive behaviors, improving emotional regulation. - Evidence shows CBT may reduce the likelihood of developing AD in depressed individuals by helping manage stress and preventing cognitive decline (González-Martín et al., 2023). *Mindfulness-based stress reduction (MBSR):* Level of evidence: moderate - Reduces stress, anxiety, and rumination by focusing on the present moment, promoting emotional resilience. - Demonstrated a delay in the onset of depression and other psychopathological conditions in the early stages of AD (Quintana-Hernández et al., 2023). *Reminiscence therapy:* Level of evidence: moderate - Uses memory recall of past events to boost emotional and cognitive well-being. - Individual or group sessions have been shown to be effective in supporting overall cognition, reducing depression, and enhancing specific aspects of quality of life in elderly individuals with AD (Cammisuli et al., 2022). *Interpersonal therapy (IPT):* Level of evidence: moderate - Targets social functioning, addressing issues like isolation and relationship difficulties that can worsen depression in AD patients. - Improved mood and better quality of life in patients with AD and depression (Miller and Reynolds, 2007).
Antidementia medication	*Cholinesterase inhibitors (ChEIs) and NMDA Receptor Antagonists -* might alleviate depression in AD patients, supported by high-level evidence from large-scale studies and clinical trials or meta-analysis *Donepezil* - shown to improve depression in AD patients (measured by BPSD screening tools). Large-scale studies (>200 patients) with 5 mg/day (1 month) and 10 mg/day (20 weeks) showed significant depression improvement, especially in severe behavioral cases (Weiner et al., 2000; Gauthier et al., 2002; Cummings et al., 2006). *Rivastigmine* - effective in alleviating behavioral depression in a 26-week placebo-controlled study (Rosler et al., 1998). *Galantamine* - modest but significant behavioral improvements in mild-to-moderate AD patients. No significant improvement in depression per Neuropsychiatric Inventory (Herrmann et al., 2005). *Memantine* - effective in improving behavioral symptoms in AD within 12 weeks (Gauthier et al., 2008). A 6-month study (399 moderate-to-severe AD patients) showed significant improvements, especially in emotional disturbances (Clerici et al., 2012). Consistent with placebo-controlled studies (Maidment et al., 2008).	*CBT for AD patients:* Level of evidence: high - Modified CBT addresses negative thoughts and behavior patterns while helping AD patients cope with cognitive decline. - Can alleviate depression and improve quality of life, enhancing coping strategies for managing symptoms of AD. Some studies suggest CBT may slow cognitive decline when combined with other treatments (Tonga et al., 2021; Orgeta et al., 2022). *Music therapy:* Level of evidence: moderate - Uses familiar or therapeutic music to reduce depression and anxiety, improving mood and cognition. - Reduces agitation and enhances overall well-being (Baruch et al., 2019; Guétin et al., 2009). *Art therapy:* Level of evidence: moderate - Encourages creativity through artistic expression to alleviate depressive symptoms and stimulate cognitive function. - Can enhance cognitive stimulation and improve emotional expression (Baruch et al., 2019).

While these medications are proven effective for major depressive disorder (MDD), their benefits in depression associated with AD may be more subtle, and the therapeutic response can vary from patient to patient. A key factor in this discrepancy lies in the distinct molecular mechanisms underlying depression in AD versus MDD (Thompson et al., 2007; Guan et al., 2022). In AD, depression is often closely linked to neurodegenerative processes such as neuronal death in the hippocampus and prefrontal cortex, which disrupt brain function and cognitive abilities. These processes lead to significant cholinergic deficits and neuropsychiatric disturbances associated with NMDA receptor dysfunction, which contribute to the symptoms of depression. Conversely, in MDD, depression is primarily associated with neurotransmitter imbalances, particularly involving serotonin, norepinephrine, and dopamine, which affect mood regulation, reward processing, and other psychological functions. Nevertheless, despite these differences, SSRIs and SNRIs can alleviate depressive symptoms in AD patients, although these medications do not directly address the underlying neurodegenerative processes specific to AD, such as cholinergic deficits and NMDA receptor dysfunction (Orgeta et al., 2017; Liu et al., 2023). Serotonergic neurons, which originate from the median and dorsal raphe nuclei, play a crucial role in regulating mood, motor activity, sleep, and aggression by innervating regions such as the limbic system and cortex, particularly the forebrain (Bartels et al., 2018). The wide-ranging projections of these neurons contribute to the antidepressant effects of serotonergic medications, which, while effective in treating depressive symptoms in AD, do not modify the fundamental neuropathological changes characteristic of the disease (Thompson et al., 2007).

In addition to pharmacological treatments for depression, antidementia medications play a significant role in treating patients with concurrent AD and depression (Table 3). Medications targeting neurodegenerative processes, such as cholinesterase inhibitors (e.g., donepezil) and NMDA antagonists (e.g., memantine), can contribute to improving both cognitive and depressive symptoms (Weiner et al., 2000; Gauthier et al., 2002; Cummings et al., 2006). These drugs may help reduce neuropsychiatric disturbances associated with AD and modulate the chemical balance in the brain, which can support the management of depressive symptoms (Hermann, 2005; Rosler et al., 1998; Maidment et al., 2008).

In addition to pharmacological treatments, non-pharmacological strategies (Table 3) have garnered increasing attention in the management of depression associated with AD. These approaches aim to address the psychological, social and neurobiological aspects of the condition, offering a complementary avenue to conventional therapies. Although antidepressants remain the first-line treatment for depression in AD, their limited efficacy and the potential side effects highlight the need for alternative, personalized interventions. One such non-pharmacological intervention is cognitive-behavioral therapy (CBT), which has been widely studied for its effectiveness in managing depressive symptoms in various populations. A recent meta-analysis has indicated that CBT may help AD patients improve coping strategies, regulate emotions and manage stress (Orgeta et al., 2022). However, its application in AD is complicated by cognitive impairments, and further research is required to adapt CBT techniques for this population, ensuring their feasibility and effectiveness. Another promising intervention is music therapy, which has been shown to improve mood, reduce anxiety, and enhance cognitive function in individuals with AD (Tonga et al., 2021). Music therapy may facilitate emotional expression and foster social interaction, thus providing both psychological and social benefits. Studies suggest that structured music interventions can promote relaxation and decrease agitation, symptoms commonly observed in AD patients. However, as with other non-pharmacological treatments, the variability in patient responses necessitates further randomized controlled trials to substantiate these findings and establish standardized protocols.

Physical activity is also gaining recognition as a valuable intervention for managing depression in AD. Regular exercise has been associated with improved mood, cognitive function, and overall well-being in older adults, including those with dementia (Baruch et al., 2019). Physical activity can enhance neuroplasticity, reduce neuroinflammation, and promote the release of neurotrophic factors, which are critical for brain health. While these benefits are promising, the optimal type, intensity, and frequency of exercise interventions remain unclear, and additional studies are needed to determine the most effective exercise regimens for AD patients. Moreover, emerging evidence supports the use of neurostimulation therapies, including transcranial magnetic stimulation (TMS) and electroconvulsive therapy (ECT), in treating depression in AD. TMS has been shown to have positive effects on mood and cognitive function, potentially by modulating brain activity in regions implicated in mood regulation. Although the use of TMS in AD is still in its early stages, a pilot randomized, double-blind, parallel, sham-controlled study suggests its potential as a non-invasive and well-tolerated option for patients with slow cognitive decline (LoBue et al., 2025). ECT, although more invasive, remains a viable treatment option for severe depression that is refractory to other therapies. Recent research into the use of ECT in AD patients is have shown symptomatic benefits in managing behavioral symptoms in individuals with dementia, particularly those with major depressive episodes (Bachu et al., 2024).

In conclusion, while non-pharmacological treatments such as CBT, music therapy, physical activity, and neurostimulation hold promise in the management of depression in AD, the evidence supporting their efficacy remains preliminary. Given the heterogeneous nature of depression in AD and the complexity of the disorder, a personalized, multimodal treatment approach that incorporates both pharmacological and non-pharmacological strategies is essential. Future research should focus on large-scale randomized controlled trials to clarify the long-term benefits, risks, and optimal protocols for these interventions. Additionally, investigating how these therapies can be integrated into routine clinical practice is vital to improving outcomes for patients with AD and comorbid depression.

## 4 Future perspectives and research directions

The treatment of depression in Alzheimer’s disease remains a multifaceted challenge, necessitating a combined effort to address the significant gaps in current understanding. While substantial progress has been made, several critical areas remain poorly understood, and future research should focus more explicitly on these unexplored or underexplored aspects.

One major gap is the neurobiological mechanisms linking depression with Alzheimer’s pathology. Specifically, the role of neurotransmitter systems such as dopamine, GABA, and neuroimmune signaling pathways remains unclear and warrants further investigation. Although some studies have explored the role of serotonin and norepinephrine, the contribution of other neurotransmitters in the pathophysiology of depression in AD is still unknown. Moreover, the interaction between neuroinflammation and cognitive decline needs further exploration, particularly regarding how inflammatory processes in the brain may exacerbate mood disturbances and accelerate Alzheimer’s progression. This knowledge could be pivotal in developing targeted therapeutic strategies aimed at modulating these pathways.

Another unresolved question is the variable efficacy of current antidepressant treatments in AD. The underlying causes of these inconsistent results, especially in relation to different stages of the disease and symptom heterogeneity, require in-depth study. Understanding the mechanisms behind the differential response to antidepressant therapies could lead to more personalized and effective treatment options. Additionally, research must focus on the development of more refined diagnostic criteria for depression in AD, as the overlap between mood disturbances and cognitive decline complicates accurate identification.

There is also an emerging need to investigate alternative pharmacological and non-pharmacological treatments that may better address the specific neurobiological features of depression in AD. While current treatments, such as selective serotonin reuptake inhibitors (SSRIs), show some promise, their inconsistent effects suggest that more targeted interventions are needed. Exploring combinations of antidepressant therapies with antidementia treatments, such as cholinesterase inhibitors and NMDA receptor antagonists, could help develop more effective, multimodal treatment regimens. Understanding the potential synergistic effects of such combined therapies could improve both cognitive and mood symptoms. Moreover, the efficacy of non-pharmacological interventions like cognitive-behavioral therapy (CBT), physical exercise, and other psychosocial approaches requires further rigorous evaluation in large-scale, randomized controlled trials. Preliminary evidence is promising, but more robust data are needed to confirm the long-term benefits and effectiveness of these therapies in diverse patient populations. Clearly defined guidelines for integrating these treatments into standard clinical practice are essential.

Lastly, the move toward personalized medicine in AD offers significant promise. Given the heterogeneity of depression in AD, integrating genetic, biomarker, and clinical data could allow for better patient stratification, leading to more effective treatments. Identifying subgroups of patients based on specific genetic predispositions, disease stages, or biomarker profiles could enhance therapeutic outcomes and reduce adverse effects.

In conclusion, while substantial progress has been made in understanding and treating depression in Alzheimer’s disease, many critical questions remain unanswered. Future research must prioritize understanding the neurobiological mechanisms of depression in AD, refining diagnostic tools, and developing more effective, personalized interventions. Addressing these gaps will not only improve the quality of care for individuals with AD but also open new avenues for therapeutic strategies that target both cognitive decline and mood disturbances.
